# The Role of Mitochondrial Dysfunction in Radiation-Induced Heart Disease: From Bench to Bedside

**DOI:** 10.3389/fcvm.2020.00020

**Published:** 2020-02-21

**Authors:** Katie Livingston, Rachel A. Schlaak, Lindsay L. Puckett, Carmen Bergom

**Affiliations:** ^1^Department of Radiation Oncology, Medical College of Wisconsin, Milwaukee, WI, United States; ^2^Cancer Center, Medical College of Wisconsin, Milwaukee, WI, United States; ^3^Department of Pharmacology & Toxicology, Medical College of Wisconsin, Milwaukee, WI, United States; ^4^Cardiovascular Center, Medical College of Wisconsin, Milwaukee, WI, United States

**Keywords:** mitochondria, radiation-adverse effects, radiation-induced cardiovascular toxicity, oxidative stress, radiation, cardiomyocyte, endothelial cell, apoptosis

## Abstract

Radiation is a key modality in the treatment of many cancers; however, it can also affect normal tissues adjacent to the tumor, leading to toxic effects. Radiation to the thoracic region, such as that received as part of treatment for breast and lung cancer, can result in incidental dose to the heart, leading to cardiac dysfunction, such as pericarditis, coronary artery disease, ischemic heart disease, conduction defects, and valvular dysfunction. The underlying mechanisms for these morbidities are currently being studied but are not entirely understood. There has been increasing focus on the role of radiation-induced mitochondrial dysfunction and the ensuing impact on various cardiac functions in both preclinical models and in humans. Cardiomyocyte mitochondria are critical to cardiac function, and mitochondria make up a substantial part of a cardiomyocyte's volume. Mitochondrial dysfunction can also alter other cell types in the heart. This review summarizes several factors related to radiation-induced mitochondrial dysfunction in cardiomyocytes and endothelial cells. These factors include mitochondrial DNA mutations, oxidative stress, alterations in various mitochondrial function-related transcription factors, and apoptosis. Through improved understanding of mitochondria-dependent mechanisms of radiation-induced heart dysfunction, potential therapeutic targets can be developed to assist in prevention and treatment of radiation-induced heart damage.

## Radiation-Induced Cardiac Disease

It has long been recognized that high-dose radiation exposure to the heart can cause cardiac dysfunction, manifesting months to decades following treatment. In 1924, radiation-induced histologic changes to the heart were first reported following radiation treatment of a patient for Hodgkin's lymphoma (1). Since that time, it has been established that therapeutic radiation to the thoracic region, for treatment of lymphomas, breast and lung cancers and pediatric malignancies can cause cardiac injuries (2). Even low doses of radiation can lead to radiation-induced heart dysfunction (RIHD), as demonstrated in epidemiologic cohorts of atomic bombing survivors and occupational exposures (1, 3, 4). Radiation can cause various structural changes to cardiac tissue, including the cardiac vasculature, leading to complications, such as pericarditis, coronary artery disease, ischemic heart disease, congestive heart failure, conduction defects and valvular dysfunction (5, 6).

Darby et al. completed a population-based case-control study of women who underwent radiotherapy for breast cancer. In this study, for every gray (Gy) of mean dose to the heart (the average mean dose was 4.9 Gy), the rate of major coronary events (myocardial infarction, coronary revascularization, or death from ischemic cardiac disease) increased by 7.4% with no upper limit. Cardiac events occurred within the first 5 years and continued several decades post-radiotherapy (7). Other studies have also examined the association between mean heart dose and cardiac events/disease, finding an ~4–16% increased risk per Gy of mean heart dose (7–9). Studies have also suggested RIHD can occur in non-small cell lung cancer patients within 2 years post-radiation exposure (8–11). In a number of lung cancer studies, mortality rates were cardiac dose-dependent, either based on mean heart dose (12) or with the percent of heart receiving 5 Gy (13), 30 Gy (13), or 50 Gy (10). In pediatric and young adult cancer patients who received cardiac radiation, there is over a 6-fold relative risk of RIHD, defined as congestive heart failure, myocardial infarction, pericardial disease, and/or valvular abnormalities (14). Cardiac events were also found to be dose-dependent, with the highest risk of events found when the mean heart dose was >30 Gy (15).

**Figure 1:**
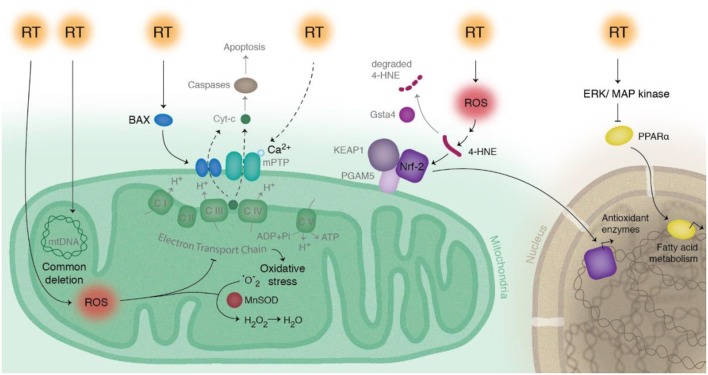
Schematic of radiation-induced effects on pathways related to mitochondria in cardiac cells. Radiation therapy (RT) directly modifies mitochondrial DNA, as seen most notably with the common deletion mutation. RT also indirectly modifies mitochondrial dysfunction by production of reactive oxygen species (ROS), leading to a disruption in the electron transport chain and increased levels of 4-HNE and increased production of antioxidant enzymes via Nrf2. Manganese superoxide dismutase (MnSOD) decreases ROS concentrations by converting superoxide (${\text{O}}_{2}^{-}$) to hydrogen peroxide (H_2_O_2_). RT decreases fatty acid energy production via activation of ERK/MAP kinase pathway, which inhibits PPAR-α. RT causes activation of Bax and release of cytochrome c, initiating the intrinsic pathway of apoptosis.

As demonstrated from these studies, cardiac exposure should be minimized when possible for radiation therapy to the thoracic region. There have been many advances in reducing cardiac exposure by improving both imaging and radiotherapy techniques (16–21). However, heart radiation exposure often remains unavoidable. There are currently no widely used methods to reverse RIHD, thus the primary way to reduce cardiotoxicity is through improved treatment planning. There is a need for preclinical studies to understand radiation-induced changes on a cellular and molecular level, with the hope of discovering new targetable pathways. Currently, there are several hypotheses on the predominant causes of RIHD, with most identified using animal models. One cause is the formation of fibrosis, distinguished by collagen deposition in and surrounding cardiomyocytes (1, 6, 22). An additional cause is macrovascular and microvascular injury, developed in a multifactorial manner by endothelial cell damage and adhesion, and activation of inflammatory and atherosclerotic responses (1, 6, 23–28). Signaling pathways, including apoptosis and mitochondrial dysfunction, have also been linked to RIHD (29, 30). This review will focus on the role of radiation-induced mitochondrial dysfunction in RIHD. The biologic pathways described in this review are illustrated in Figure 1, and the discussed clinical and preclinical studies are summarized in Table 1.

**Table 1:** Summary of studies investigating the role of mitochondria in RIHD.

**References**	**Tissue type/Study subject**	**Methods/intervention**	**Radiation**	**Result**
Burch et al. (31)	*in vivo*; human cardiomyocyte	Electron microscopy of irradiated tissue of mediastinum	52 Gy, unknown fractionation	Mito swollen; reduced and disorganized cristae; fused double membrane
Khan (32)	*in vivo*; rabbit myocardial cells	Electron microscopy of irradiated heart tissue	10 or 13 Gy, single dose	Altered mito structure 48 h post-RT exposure
Prithivirajsingh et al. (33)	*in vitro*; human cell lines (dermal fibroblasts, AT, KSS, DNA glioblastoma, and colon carcinoma cell lines)	Evaluation of common deletion	Cesium-137, 4.17 Gy/min, total of 5, 10 or 20 Gy	Increased levels of common deletion 72 h post-RT; dose-independent
Azimzadeh et al. (34)	*in vivo*; C57BL/6 mice; cardiac tissue protein lysates	Proteomic analysis of irradiated mito proteins	TBI, 3 Gy, single dose	5 and 24 h post-RT—increased levels of proteins involved in oxidative phosphorylation (ATP synthase, NADH dehydrogenase, cytochrome c oxidase)
Barjaktarovic et al. (35)	*in vivo*; C57BL/6N mice; isolated cardiac mito	Mito proteomic and functional analysis of low dose RT localized to heart (4 weeks)	0.2 or 2 Gy, single dose	4 weeks post-RT, 2 Gy (functional and proteomic changes); 0.2 Gy functional changes only)
Barjaktaroic et al. (36)	*in vivo*; C57BL/6N mice; isolated cardiac mito	Mito proteomic and functional analysis of late effects (40 weeks) of low dose RT localized to heart	0.2 or 2 Gy, single dose	40 weeks post-RT: 2 Gy (functional and proteomic changes); 0.2 Gy (no significant effect)
Boerma et al. (37)	*in vivo*; *Gsta4*-null vs. WT mice; cardiac tissue	Analysis of cardiac function and proteomics following local heart RT	18 Gy, single dose	Reduced CO, SV and EF in WT. Increased levels of PGAM5 and Nrf2 in *Gsta4*-null-mice
Azimzadeh et al. (38)	*in vivo*; C57BL/6 mice; cardiac tissue protein lysates	Analysis of PPAR-α activity following local radiation to the heart	8 or 16 Gy, single dose	PPAR-α inactivated post-RT with increased FFA, decreased mito complexes I, III, V
Azimzadeh et al. (4)	*in vivo*; human cardiomyocytes	Epidemiologic proteomic analysis following chronic occupational exposures	100 mcGy−5 Gy, chronic exposure	Dose-dependent increase phosphorylation of PPAR-α and decrease in mito complex I and III and Nrf2
Salata et al. (39)	Wistar rats; left ventricular cardiac tissue	Analysis of apoptotic factors 5 months post-cardiac RT	20 Gy, single dose	Increased expression Bax/Bcl2, increased apoptotic nuclei
Sridharan et al. (40)	Male Sprague-Dawley rats; isolated left ventricular cardiac mito	Analysis of time course of RT mito apoptotic changes (at 2 h−9 months post-RT)	3–21 Gy, single dose	Bax/Bcl2 ratio elevated (6 h−6 months). Apoptotic nuclei (6 and 24 h and 2 weeks) Increased calcium-induced swelling/ MPT susceptibility (6 h−9 months)
Ferreira-Machado et al. (41)	Female Wistar rat cardiomyocytes	Analysis of caspase activity 13 months post heart RT	15 Gy, single dose	Cleaved/activated caspase at 13 months post-radiation
Franco et al. (42)	HEK-293 cells	Analysis of GRK activity post-RT	4 Gy, single dose	Overexpression of GRK preserved mito morphology, maintained membrane potential and enhanced respiration (3–8 h post-RT)

*RT, Radiation therapy; mito, mitochondria; AT, ataxia telangiectasia; KSS, Kearns Sayre Syndrome; MnSOD, manganese superoxide dismutase; TBI, total body irradiation; WT, wildtype; CO, cardiac output; SV, stroke volume; EF, ejection fraction; FFA, free fatty acids, MPT, membrane permeability transition; RT, radiation therapy*.

## Mitochondria and Oxidative Stress

The role of cardiomyocyte mitochondria is critical to cardiac function, with each cardiomyocyte having abundant mitochondria that make up ~30% of cell volume (43, 44). Mitochondria have a role in stress responses, cell death, and metabolic processes. They are essential for energy production, which is created by products of glycolysis and fatty acid metabolism via oxidative phosphorylation in the respiratory chain, yielding reactive oxygen species (ROS) biproducts, such as superoxide, peroxide and hydroxyl radicals. Wang et al. reviewed the normal mitochondrial mechanisms, as well as manners in which equilibrium can be interrupted in the heart after radiation. In homeostasis, ROS facilitate cellular functions, including immune responses, signal transduction and apoptosis. ROS can be neutralized by antioxidants when their concentrations are in excess (29). If this highly regulated process is disrupted, increased production or decreased removal of ROS can lead to cellular and DNA damage (29).

Stress-induced mitochondrial damage can cause a loss of mitochondrial membrane potential, leading to the mitochondria undergoing either fission or fusion. Fission helps mitigate stress by fusing parts of damaged mitochondria with normal mitochondria and is regulated by proteins including Opa1 and Mitofusin-1 and−2 (Mfn-1 and Mfn-2). Fission is needed to help create new mitochondria, but also serves as quality control through facilitating apoptosis during high levels of cellular stress. Fission is mediated by proteins including Drp1 and Mft (45, 46). If damaged mitochondria need to be eliminated, kinase PINK1 yields as a sensor of mitochondrial damage and signals to induce mitophagy (46). These complex dynamic mitochondrial processes are imperative in cardiomyocytes, as these cells obtain more than 90% of their energy from mitochondrial respiration (35, 40). This makes mitochondria within these energy-demanding cells an ideal study model to characterize mitochondrial changes that occur after radiation exposure.

It was discovered in the late 1960s that radiation can drastically alter the structural appearance of mitochondria, both short- and long-term. Seven years after high dose radiation (52 Gy) to the mediastinal region in a human patient, electron microscopy revealed cardiomyocyte mitochondria that were variably swollen with decreased number and disorganization of cristae, often with fused outer double membranes (31). Changes in mitochondrial structural integrity occurred as early as 48 h following exposure in rabbit myocardial cells which had received a single dose of either 10 or 13 Gy (32). These findings have led to additional studies on the role of mitochondrial function in RIHD.

On a molecular level, ionizing radiation directly modifies DNA, including single- and double-stranded breaks, base damage, and cross-links, all of which can lead to cell death if not repaired properly. Indirectly, radiation can lead to ROS formation, which can cause cellular stress and death (1). Mitochondrial DNA (mtDNA) is a major radiation target because it lacks the protective effects of histones (47). In addition, it is generally repaired less efficiently than nuclear DNA (48) and has a mutation rate 10–1,000 times higher than nuclear DNA, making it an ideal model to study the mutational effects of radiation (33, 48, 49). This has been most notably reflected with the mutation called common deletion—a 4,977 base pair deletion within mtDNA that has become a marker for oxidative damage. Increased levels of the common deletion have been noted in human cardiac cells undergoing oxidative stress secondary to atrial fibrillation (50). Other studies have noted radiation-induced development of common deletion within human cell lines, both in low (0.1 Gy) and therapeutic doses (>1 Gy) (33, 48, 51), although no studies to date have specifically analyzed radiation-induced common deletion in cardiomyocytes. All mtDNA genes are essential for the biogenesis and function of mitochondria, so mutations leading to altered overall gene expression would be expected to cause a deficiency in energy metabolism and enhanced production of ROS, leading to oxidative stress (40).

Alterations in proteins that affect ROS generation and oxidative stress may also enhance RIHD. Manganese superoxide dismutase (MnSOD), a mitochondrial matrix enzyme that protects against oxidative stress by converting superoxide to H_2_O_2_, can decrease cardiac injury severity. Mice deficient in MnSOD died within the first 10 days of life and exhibited dilated cardiomyopathy, among other abnormalities. A study by Nojiri et al. generated cardiac-specific MnSOD-deficient mice, and these mice developed congestive heart failure with severe cardiac muscle degeneration and significantly reduced ATP production, demonstrating that alterations in enzymes important for maintenance of ROS levels can lead to oxidative stress-dependent heart disease (52). Other studies have shown that MnSOD can play an important role in ischemia-reperfusion cardiac injury as well (52).

G protein-coupled receptor kinase (GRK) is another protein reported to regulate ROS in response to stress. Studies have shown data that GRK may act both a protector against and a promotor for death following ischemic injury (45, 53). Removal of cardiac-specific GRK2 has been linked to embryonic cardiovascular development, adult cardiac dilatation, early atherosclerosis and inhibited angiogenesis in mice (42, 54–57). Franco et al. evaluated cell cultures with knockdown or overexpressed GRK2 3–8 h after exposure of a single dose of 4 Gy. Knockdown of GRK caused morphologic mitochondrial changes, reduced membrane potential and reduced mitochondrial function (42). The overexpression of GRK2 protected mitochondria from radiation damage (42). Furthermore, it was demonstrated that in the presence of heat shock proteins, GRK2 interacts with mitofusins (MFN-1 and MFN-2), key regulators of mitochondrial fission and fusion (42, 58). Additional studies are required to understand the chronic response of GRK to radiation, but GRK-related pathways may be important targets to drive mitochondrial protection from radiation-induced damage.

Several other studies have noted radiation-induced mitochondrial changes in terms of oxidative stress and respiratory capacity in mice (34). These changes have been noted h to months following radiation exposure. Five and 24 h after 3 Gy total body irradiation, C57BL/6 mice had immediate changes in cardiac structure and function. On murine cardiac tissue proteomic analysis, mitochondrial proteins represented the protein class most sensitive to radiation, with increased levels of proteins involved in oxidative phosphorylation, including ATP synthase, NADH dehydrogenase and cytochrome *c* oxidase (34).

Chronic low dose exposure of ionizing radiation can cause heart disease, as noted in atomic bomb survivors and nuclear power industry workers (3, 4, 59). As previously mentioned, doses as low as 0.1 Gy caused accumulation of the common deletion in human cell lines (51). However, other studies have shown minimal effect with these low doses. Barjaktarovic et al. studied C57BL/6N mice that received either 0.2 Gy, 2 Gy to the heart or sham radiation. Four weeks post-exposure, cardiac mitochondria were examined for proteomic and functional alterations. After 2 Gy, both functional and proteomic alterations were observed. Proteomic analysis revealed a total of 25 downregulated proteins, in three biological areas: oxidative phosphorylation, pyruvate metabolism and cytoskeletal structures. Functional impairment was reflected as partial deactivation of mitochondrial Complex I and III, decreased succinate-driven respiratory capacity, increased ROS levels and enhanced oxidation of mitochondrial proteins. At the lower dose (0.2 Gy), only proteomic changes were identified, suggesting a dose-dependence of mitochondrial dysfunction after cardiac radiation (35). This group then investigated the late cardiac effects at 40 weeks post-exposure, at which time respiratory capacity of the mitochondria was still reduced after 2 Gy. This suggests that radiation can cause non-transient alterations of oxidative stress in mitochondria (36).

## Glutathione S-Transferase Alpha 4 (GSTA4-4)/Nrf2 Pathway

Another method of measuring oxidative stress is by quantifying downstream transcription factors. During the process of lipid peroxidation, 4-hydroxynonenal (4-HNE) concentrations increase, which directly activates nuclear factor erythroid 2 [NF-E2]-related factor 2 (Nrf2, gene name *NFE2L2*), a transcription factor that targets a number of antioxidant proteins. Nrf2 is a redox-sensitive factor that controls oxidative responses within cells and has a role in endothelial function and cardiac protection (22, 60). The repressor protein Keap1 binds and sequesters Nrf2, promoting Nrf2 ubiquitin-mediated degradation. The protein phosphoglycerate mutase family member-5 (PGAM5) is attached to the mitochondrial membrane and can form a complex with Keap1 and Nrf2 (37, 61, 62). Under oxidative conditions, Nrf2 is released from the complex, allowing nuclear accumulation of Nrf2 (60, 62). The role of Nrf2 was indirectly verified by the impact of glutathione S-transferase alpha 4 (GSTA4-4), which is an enzyme that removes 4-HNE. A study with *Gsta4-*null mice noted enhanced resistance to the cardiotoxic effects from doxorubicin, suggesting a compensatory mechanism may have caused cardiac protection (37). Boerma et al. conducted a similar study with the *Gsta4*-null mice with local heart irradiation to a total dose of 18 Gy. Six months post-radiation exposure, the wild-type mice had reduced cardiac output, stroke volume and ejection fraction, with associated increased levels of cardiac troponin-I levels when compared to the *Gsta4*-null mice. Additionally, the *Gsta4*-null mice had increased mRNA levels of PGAM5 and Nrf2. Nrf2 was also significantly elevated in the sham-irradiated *Gsta4*-null mice when compared to wild-type mice. When comparing the levels of 14 different Nrf2 target genes, none were significantly elevated in wild-type irradiated mice; seven genes were significantly elevated in irradiated *Gsta4-*null mice compared to non-irradiated *Gsta4-*null mice, suggesting a stronger activation of the Nrf2 pathway in the irradiated *Gsta4-*null mice (37).

Another study found that genes on rat chromosome 3 can alter RIHD by using consomic rat strains to identify genetic variants that cause differences in cardiac radiosensitivity. One week after 24 Gy of localized cardiac radiation, changes in expression of numerous gene pathways, including mitochondrial function, were seen between the sensitive and resistant rat hearts. Nrf2 was found to be an upstream regulator of many of the enriched pathways (61). Other preclinical investigations have studied the protective role of the Nrf2 pathway on radiation injury to cardiomyocytes and other cell lines, including embryonic fibroblasts and breast and lung epithelial cells (37). These results taken together suggest Nrf2 and GSTA-4 pathways may be promising targets for reducing mitochondrial dysfunction from cardiac radiation exposure.

## Peroxisome Proliferator-Activated Receptor-α (PPAR-α)

Cardiac muscle preferentially relies on fatty acid energy production via oxidative phosphorylation over glucose metabolism. Peroxisome proliferator-activated receptor-α (PPAR-α) is a highly expressed transcription factor in tissues with elevated lipid metabolic turnover, including cardiac tissue. When PPAR-α is downregulated, lipid metabolism is impaired, as noted in PPAR-α-null mice (38), suggesting PPAR-α regulates energy equilibrium. When C57BL/6 mice were exposed to 8 or 16 Gy of cardiac radiation, PPAR-α was phosphorylated by ERK-MAPK causing decreased transcriptional activity, leading to increased free fatty acid levels and reduced levels of mitochondrial complexes I, III and V (38). The finding that radiation-induced PPAR-α alterations cause decreased expression of energy metabolism and mitochondrial respiration-related genes has been corroborated in human subjects with chronic radiation exposure. In the 1940s, the Mayak Production Association built a nuclear facility in Russia, where workers were chronically exposed to incidental radiation during their occupational duties. Epidemiologic cohorts were analyzed to identify workers who died from heart disease and individual dosimetric monitors were used to determine radiation exposure. Studies in this cohort of individuals found significant increases in heart disease associated with total external gamma-ray doses, even after adjusting for confounding factors, such as smoking exposure (63–65). In a separate study, the protein expression from post-mortem heart samples were examined from a subset of workers exposed only to external gamma rays who had a diagnosis of ischemic heart disease and a primary cause of death of ischemic heart disease. Total doses of external exposure in this cohort ranged from 100 mcGy to more than 5 Gy. Proteomic analysis from 29 individuals identified a dose-dependent increase in phosphorylation of PPAR-α with a corresponding dose-dependent decrease in mitochondrial proteins, such as complexes I, III, and Nrf2 (4). PPAR-α has already shown an effect in other cardiovascular risk factors in preclinical and clinical studies (for dyslipidemia and diabetes mellitus) and is now an encouraging targetable agent for potential mitigation of RIHD (6, 66).

## Mitochondria and Apoptosis

Mitochondria are critical for some methods of programmed cell death, or apoptosis. For the intrinsic pathway, the inner and outer mitochondrial membranes must be permeabilized to release apoptotic factors, such as cytochrome *c*. The Bcl-2/Bax family of proteins help regulate and stabilize the membranes and govern the predilection for mitochondrial membrane permeabilization (29, 67). Once Bax is activated, it translocates from the cytoplasm to the mitochondrial membrane, where it can induce membrane permeability transition (MPT). MPT is characterized by mitochondrial swelling, depolarization of the membrane and uncoupled oxidative phosphorylation. It can also be induced by calcium influx and ROS. Radiation may also cause mitochondrial-mediated apoptosis due to the close association between the endoplasmic reticulum and mitochondria. When cardiomyocytes are irradiated, the endoplasmic reticulum releases a flux of excess calcium ions (Ca^2+^) that facilitate permeabilization of mitochondria (29). Animal studies have shown increased levels of the Bax/Bcl2 ratio expression following irradiation. In one analysis, increased expression levels of Bax/Bcl2 and increased apoptotic nuclei were seen in Wistar rats 5 months after cardiac radiation (20 Gy), with an associated increase of fibrotic tissue and cardiomyocyte hypertrophy (39). Sridharan et al. investigated the time course of radiation-induced changes to mitochondria in rats sacrificed 2 h to 9 months following a single dose of radiation, ranging from 3 to 21 Gy. Levels of Bax and Bcl2 were significantly increased by 6 h post-exposure. The Bax/Bcl2 ratio was elevated from 6 h to 6 months after irradiation, but not significantly elevated at 9 months. These findings were associated with apoptotic nuclei at 6 and 24 h and 2 weeks following radiation (40). One study identified cleaved caspase 3, an apoptosis activator, as late as 13 months post local heart radiation (15 Gy) to Wistar rats (40, 41). Additionally, the study completed by Sridharan et al. noted increased radiation-induced susceptibility to MPT, measured by increased calcium-stimulated mitochondrial swelling. At time points ranging from 6 h to 9 months post-radiation, in a dose-dependent manner, irradiated cardiac mitochondria were more susceptible to calcium-induced swelling. Previously, studies have noted only a transient depolarization of the mitochondrial membrane potential and MPT after radiation. This suggests that radiation may cause an enhancement in the susceptibility of MPT and pore opening in mitochondria to subsequent stressors (40).

## Endothelial Cell Mitochondria

Radiation exposure can induce endothelial cell activation, shifting endothelial cells into a pro-inflammatory state. When exposure is repeated or prolonged, the endothelium can alter its protective physiology, which can lead to exhaustion and a decrease in vascular function. This endothelial dysfunction leads to decreased vascular tone, inflammation and atherosclerosis, all of which may contribute to cardiovascular disease (28). Concentrations of mitochondria are relatively low in endothelial cells compared to cardiomyocytes and mitochondria produce a lower portion of total endothelial cell energy. However, endothelial cell mitochondria have been found to play important roles in cellular signaling (28). Radiation-induced endothelial cell mitochondrial dysfunction may contribute to RIHD, though data on this topic is currently limited. Endothelial cell functions that can be altered by radiation include Ca^2+^ regulation, apoptosis and oxidative stress signaling. Radiation-induced release of Ca^2+^ from the endoplasmic reticulum leads to increased mitochondrial Ca^2+^ uptake, yielding membrane swelling and release of apoptotic factors (29). However, Ca^2+^ plays numerous roles in signaling pathways and intracellular functions that theoretically may be affected by radiation (e.g., inner membrane calcium uniporter, mitochondrial Ca^2+^ activation of dehydrogenase enzymes and ATP synthase and TNF-α-induced inflammation). These concepts have just begun to be addressed preclinically in the setting of radiation exposure (28).

Baselet et al. illustrated that dysregulation of the Bcl2 pathway (intrinsic apoptosis pathway) yields endothelial inflammation, apoptosis and senescence, all of which are coupled with atherosclerotic development (28). Along similar lines, cells can undergo senescence, the irreversible arrest of endothelial cell renewal, after extensive cell division or exposure to stressors, including radiation. Previous *in vitro* and *in vivo* studies have noted evidence of endothelial cell senescence following local radiation exposure. In human umbilical vein endothelial cells (HUVECs), mitochondrial membrane potential was altered 2 days after irradiation with 1.5, 4, and 10 Gy. The membrane potential returned to baseline levels at days 5 and 6 with 1.5 and 4 Gy, respectively; however, mitochondrial activity remained reduced in cells irradiated with 10 Gy (28). The underlying mechanisms of radiation-induced senescence are not fully established, though mechanisms may involving the p53-p21 and the IGF1-PI3K-Akt/mTOR pathways that may be attributable to the downregulation of Silent Information Regulator-1 (SIRT1) (68). SIRT1 is a NAD-dependent deacetylase that regulates many proteins involved in mitigating oxidative stress, and although its relationship to RIHD has not been explored, SIRT1-deficiency increased the sensitivity of thymocytes to apoptosis (69). Similar to cardiomyocytes, studies of *in vitro* endothelial cells noted increased production of ROS 24–72 h post-radiation exposure (5–20 Gy) (28). In addition, Nrf2 upregulation has also been implicated in oxidative stress-induced endothelial dysfunction (1, 28). Furthermore, proteomic data on C57BL/6 mice receiving 8 or 16 Gy of local heart irradiation revealed expression of proteins associated with mitochondrial dysfunction within endothelial cells (70).

## Conclusion

Radiation exposure to the thoracic region can cause a variety of cardiac injuries. Numerous preclinical animal and cell models have studied the mechanisms behind RIHD, though these are not yet fully elucidated. Here we have reviewed several factors related to radiation-induced cardiomyocyte and endothelial cell mitochondrial dysfunction, including mtDNA mutations, oxidative stress, alterations in various transcription factors and apoptosis. These factors ultimately play a role in the complex mitochondrial dynamics that can change the fate of cardiac cells. Through further understanding of mitochondria-dependent mechanisms of RIHD, potential therapeutic targets can be developed to prevent and/or treat radiation-induced heart damage.

## Author Contributions

All authors contributed to conception and design of the review, wrote sections of the manuscript, contributed to manuscript revision, read, and approved the submitted version.

### Conflict of Interest

The authors declare that the research was conducted in the absence of any commercial or financial relationships that could be construed as a potential conflict of interest.
